# Linkages between soil-specific enzyme activity and microbial necromass carbon: implications for ecological restoration

**DOI:** 10.3389/fmicb.2026.1860192

**Published:** 2026-06-12

**Authors:** Xiaohan Zhou, Ruiyang Wang, Xuying Hai, Hongwei Xu, Jiao Li, Li Zhang

**Affiliations:** 1 Forest Ecology and Conservation in the Upper Reaches of the Yangtze River Key Laboratory of Sichuan Province, Sichuan Mt. Emei Forest Ecosystem National Observation and Research Station, College of Forestry, Sichuan Agricultural University, Chengdu, China; 2 School of Forestry and Grassland Science, Ningxia University, Yinchuan, China

**Keywords:** amino sugars, degeneration, desertification, soil microbial activity, vegetation restoration

## Abstract

Ecological restoration can effectively boost soil fertility and microbial activity in degraded ecosystems. Yet, its impact on the relationship between soil-specific enzyme activity (SSEA) and microbial necromass carbon in barren sandy soils is still poorly understood. Therefore, we aimed to analyze the effects of ecological restoration on the SSEA per unit of soil organic carbon (SOCE) and SSEA per unit of microbial biomass carbon (MBCE), as well as their correlation with the necromass accumulation coefficient (AC). To this end, we collected and analyzed soil samples from the grazing land (control) and restored grassland, scrubland, and forestland in a typical degraded sandy land. Compared with grazing land, restored grassland and scrubland had lower SOCE levels of β-1, 4-glucosidase (BG), β-D-cellobiosidase (CBH), L-leucine aminopeptidase (LAP), and acid phosphatase (AP), whereas restored forestland had a higher SOCE level of β-1, 4-N-acetylglucosaminidase (NAG). Additionally, restoring to scrubland and forestland increased the MBCE of BG, CBH, NAG, LAP, and AP, while restoring to grassland, scrubland, and forestland increased the geometric mean of enzyme activity (GMEA) by 59.5, 46.8, and 108.2%, respectively, compared with grazing land. The SOCE of BG, LAP, and AP showed quadratic relationships with SOC, and the MBCE of BG, CBH, LAP, and AP was positively correlated with necromass AC. SOCE and MBCE were substantially affected by ecological restoration. Our results emphasize that SOCE can be used as an indicator of SOC, and MBCE can be used to predict changes in microbial necromass during vegetation restoration.

## 1 Introduction

Soil degradation refers to the process by which the internal structure and physical and chemical properties of soil are damaged, resulting in the loss of its original functions (Zhou et al., 2025). Land desertification, as one form of soil degradation, manifests as soil coarsening and a decline in fertility resulting from the disruption of soil texture and the loss of nutrients (Reichert et al., 2016). This not only leads to a substantial amount of land resource waste but also affects the development of agriculture, animal husbandry, and ecological construction (Reynolds et al., 2007). In response to this challenge, vegetation restoration is considered an effective approach to improving soil structure, restoring soil fertility, and enhancing the biological functions of degraded ecosystems (Jiang et al., 2014; Zhou et al., 2025). Studies have shown that ecological restoration measures can not only significantly improve soil physicochemical properties but also effectively reverse the process of land desertification (Zheng et al., 2024).

Soil enzyme activity is a key index for assessing soil fertility in ecosystem restoration (Qiao et al., 2025; Somchanh et al., 2026). Soil enzymes and plant communities interact and regulate each other during the restoration of vulnerable ecosystems (Zuccarini et al., 2023). Soil enzymes affect plant productivity by influencing soil fertility (Qin et al., 2020). Conversely, plants supply nutrients and energy to microbes via roots and litter, regulating enzyme secretion and activity (Brackin et al., 2013). However, traditional measures of soil enzyme activity—namely, soil absolute enzyme activity (per unit mass of soil), have limitations when characterizing soil nutrient cycling and do not fully reflect soil carbon turnover processes (Yu et al., 2019; Xu et al., 2020). As sensitive indices, soil-specific enzyme activity (SSEA) [expressed in units of soil organic carbon (SOCE) or microbial biomass carbon (MBCE)] has been widely used to reflect changes in soil organic matter dynamics and microbial enzyme production efficiency (Qu and Hai, 2025; Liu et al., 2017).

Extensive studies have examined the impacts of ecosystem degradation and restoration on soil enzyme activity. For example, Ferreira Araujo et al. (2013) analyzed microbial characteristics of soil under different degradation intensities in northeastern Brazil and found that moderate and severe degradation reduced soil enzyme activity. Based on global-scale data, Xu et al. (2023) and Breidenbach et al. (2022) determined that ecosystem degradation caused by grazing reduced soil carbon and phosphorus enzyme activities. The decrease in soil enzyme activity due to ecosystem degradation is mainly attributed to soil structure destruction, vegetation reduction, and soil nutrients decrease (Liu et al., 2023). However, ecological restoration promotes vegetation restoration and soil development and increases soil nutrients, providing abundant substrates for microbial growth and reproduction (Graham and Knelman, 2023; Araujo et al., 2024). Thus, enzyme production efficiency is enhanced (Sun et al., 2022). Moreover, a recent meta-analysis emphasized that ecological restoration increases microbial activity (Sha et al., 2023). However, the effects of ecological restoration on enzyme activity may vary among different restoration approaches. For instance, Wang G. et al. (2024) observed that enzyme activity declined initially before rising with extended restoration time, emphasizing that restoring abandoned farmland is key to improving soil structure and enzyme activity. Tian et al. (2024) studied forests at different succession stages (10, 20, 30, and 40 years) and found that ecological restoration increased enzyme activity. However, studies on SSEA have mainly focused on land use changes (Yu et al., 2019; Guan et al., 2022), urea addition (Shan et al., 2015), paddy rice cultivation (Raiesi and Beheshti, 2014), the application of composted sewage sludge (Liu et al., 2017), and grassland succession (Xiao et al., 2021). Mechanisms linking ecological restoration to SSEA are poorly understood, and systematic studies are needed to clarify if its effects vary by vegetation type.

The soil carbon pool is huge, and boosting its carbon sequestration capacity is a key focus in ecological development (Deng et al., 2014). Increasing SOC enhances soil structural stability (Vogel et al., 2014) and reduces erosion risk (Doetterl et al., 2016). Microorganisms are key to SOC formation and transformation (Delgado-Baquerizo et al., 2023). While scientists once thought microbes promoted SOC accumulation mainly by decomposing litter and roots (Liang et al., 2017), growing evidence shows their primary contribution is via microbial necromass (Liang et al., 2019). Defined as organic complexes from decomposed microbial cells and fragments (Craig et al., 2022), necromass plays a critical role in SOC accumulation (Camenzind et al., 2023). An in-depth study of the factors driving the accumulation of SOC and microbial necromass will elucidate the soil carbon sequestration potential and provide guidance for developing targeted measures to improve soil quality (Bai and Cotrufo, 2022). Past studies have emphasized that SOCE and MBCE affect soil carbon stability and carbon component accumulation (Xu et al., 2020). However, it remains unclear whether SOCE and MBCE affect the accumulation of SOC and microbial necromass carbon.

Overall, the effects of ecological restoration on SSEA remain poorly understood. Therefore, this study aimed to explore how ecological restoration affects SOCE and MBCE, and clarify their correlations with microbial necromass, and identify the optimal restoration strategy to enhance SOCE and MBCE. To achieve these goals, we collected and analyzed soil samples from grazing lands and restored ecosystems (grasslands, scrublands, and forests) in typical degraded sandy areas of the Loess Plateau. We hypothesized that: (1) ecological restoration significantly increases SOCE and MBCE, and the positive effects of restoring land to scrubland and forestland are higher than those of restoring land to grassland; and (2) SOCE and MBCE are significantly correlated with microbial necromass carbon and SOC and can be used as important predictors of microbial necromass and SOC accumulation during ecological restoration.

## 2 Methods

### 2.1 Study area

The study area was in the eastern desert of Ningxia Hui Autonomous Region (Supplementary Figure S1), a typical degraded sandy land undergoing ecological restoration. The soil type is eolian sand. The climate type is continental monsoon, which is characterized by low and concentrated rainfall and a high evaporation rate. The average annual precipitation and temperature are 250–350 mm and 6.0 °C–8.5 °C, respectively. Overgrazing has caused serious degradation in the study area, and grazing bans and vegetation restoration were implemented in 2015. The main source of surface water resources in the study area is precipitation, but the lack of rainfall and poor water and nutrient conservation have hindered ecological restoration in this area. However, after 10 years of restoration, the study area showed good vegetation recovery and typical grassland, scrubland, and forestland communities, indicating its suitability as a natural research site for ecological restoration.

### 2.2 Experimental design

Three vegetation types (recovery time of approximately 10 years) were selected in the study area: grassland, scrubland, and forestland. The grazing land was used as the control. Specifically, three plots of grassland and scrubland were selected, and nine plots of forestland (*Populus alba, Pinus tabuliformis,* and *Robinia pseudoacacia*) were selected. The spacing between plots was >100 m. Two quadrats were selected from each plot (the grassland quadrats were 1 m × 1 m, the scrubland quadrats were 2 m × 2 m, and the forestland quadrats were 10 m × 10 m).

### 2.3 Soil sample collection and analysis

We collected 36 soil samples (0–20 cm depth) from all quadrats, dividing each into three subsamples: two were stored at 4 °C and −20 °C for analyzing soil enzyme activity, microbial biomass, and microbial community structure, respectively. The remaining part of the soil was sifted, naturally air-dried, and ground for the determination of soil chemical properties. Soil basic chemical properties, microbial biomass, enzyme activity, and microbial community results are listed in Supplementary Table S1 and Supplementary Figure S2.

The soil pH, soil organic carbon (SOC), total nitrogen (TN), and total phosphorus (TP) were determined using the pH meter, the H_2_SO_4_-K_2_Cr_2_O_7_ oxidation method, the Kjeldahl method, and the ammonium molybdate method, respectively. Microbial biomass carbon (MBC) and microbial biomass nitrogen (MBN) were determined using the fumigation extraction method as described by Xue et al. (2017) and Xu et al. (2020). Enzyme activities, including β-1,4-glucosidase (BG), β-D-cellobiose hydrolase (CBH), β-1,4-N-acetylglucosaminidase (NAG), L-leucine aminopeptidase (LAP), and acid phosphatase (AP), were measured using 96-microplate enzymic fluorescence assays (Saiya-Cork et al., 2002): CH_3_COOH buffer was added to 1.0 g of soil, mixed thoroughly, and incubated; fluorescence was then measured using a 96-well plate fluorometer. The microbial community structure was determined by Rhonin Biosciences in Chengdu, China.^1^ The amino sugars were determined by Baihui Organisms^2^ using gas chromatography.

### 2.4 Calculations and statistical analysis

The soil-specific enzyme activity (SSEA) of SOCE and MBCE were calculated using the following formulas (Trasar-Cepeda et al., 2008):

$$\text{SOCE}=\frac{\text{Soil absolute enzyme activity}\;}{\mathrm{SOC}}$$

$$\text{MBCE}=\frac{\text{Soil absolute enzyme activity}\;}{\mathrm{MBC}}$$

The soil enzyme activity coefficient, geometric mean of enzyme activity (GMEA), and necromass accumulation coefficient (AC) were calculated as follows (Qu and Hai, 2025; Fu et al., 2025):

$$\text{Soil enzyme activity coefficient}=\frac{\text{Carbon}/\text{Nitrogen enzyme activity}}{\mathrm{MBC}/\mathrm{MBN}}$$

$$\text{GMEA}=\sqrt[{5}]{\mathrm{BG}\times \mathrm{CBH}\times \mathrm{NAG}\times \mathrm{LAP}\times \mathrm{AP}}$$

$$\text{Necromass}\;\mathrm{AC}=\frac{\text{Microbial necromass carbon}}{\mathrm{MBC}}$$

The detailed methods of microbial necromass carbon were described in Qu and Hai (2025). One-way analysis of variance was performed to test the differences among different indices (*p* < 0.05). Regression analysis was used to examine the correlations among SSEA, necromass AC, and SOC. The Mantel test and redundancy analysis (RDA) method were used to explore the effects of chemical properties, microbial biomass, enzyme activity, and bacterial and fungal diversity on SSEA.

## 3 Results

### 3.1 Dynamics of SOCE

**Figure 1:**
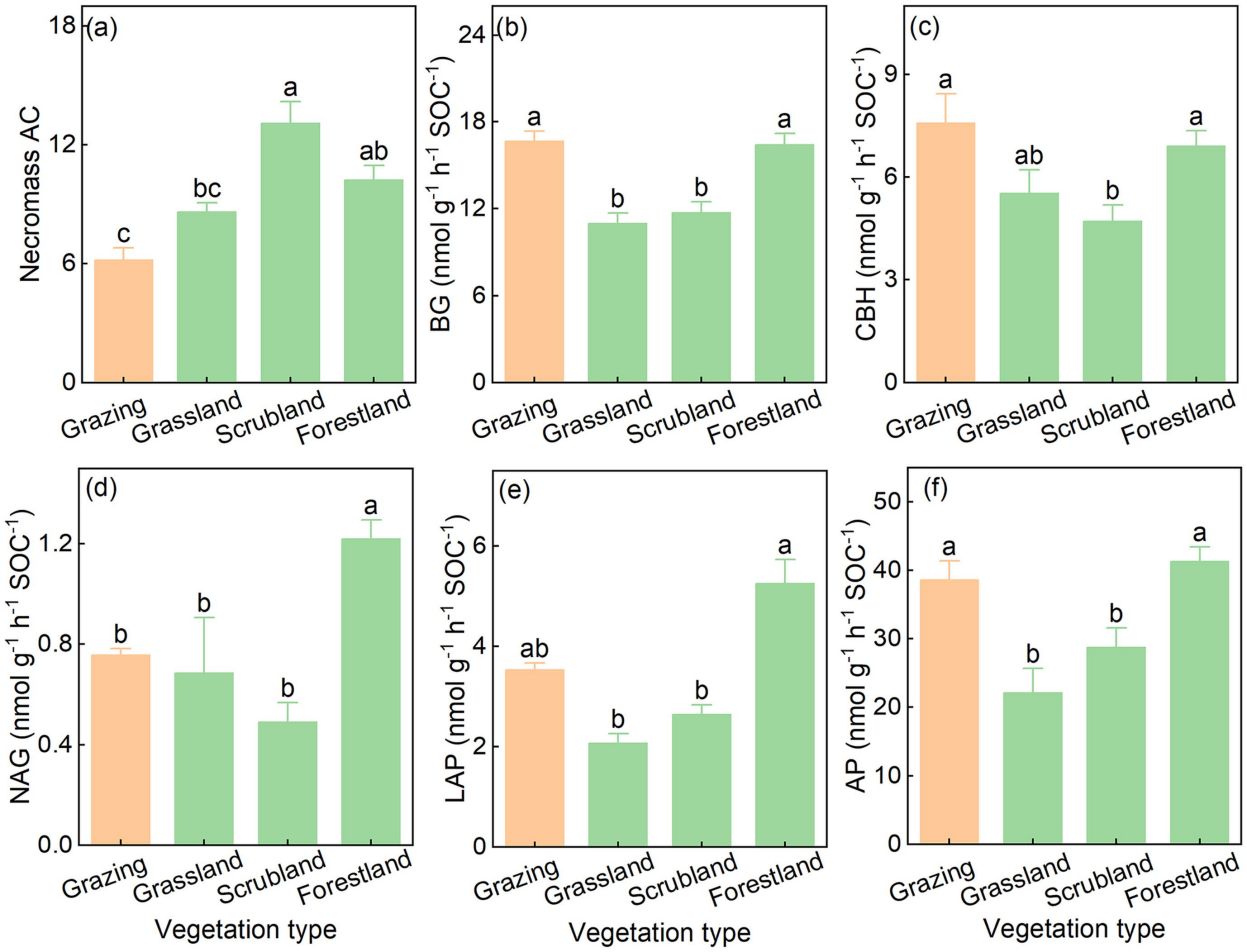
Effects of vegetation restoration on soil microbial necromass AC (a), and SOCE of BG (b), CBH (c), NAG (d), LAP (e), and AP (f). Necromass AC, microbial necromass carbon accumulation coefficient; SOCE, soil-specific enzyme activity per unit of soil organic carbon; BG, β-1, 4-glucosidase; CBH, β-D-cellobiosidase; NAG, β-1, 4-N-acetylglucosaminidase; LAP, L-leucine aminopeptidase; AP, acid phosphatase. Different letters indicate significant differences at *p* < 0.05.

**Figure 2:**
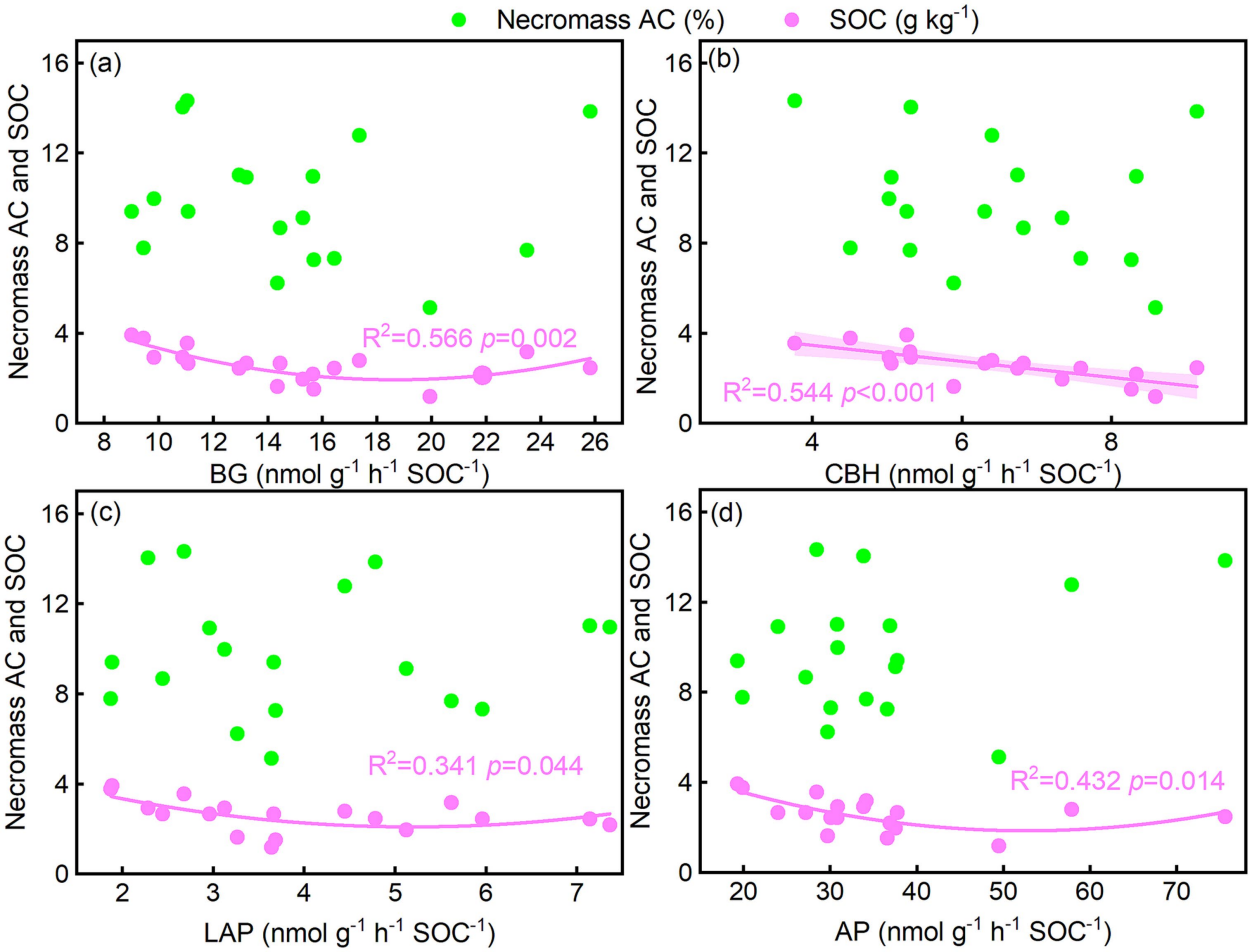
Regression for SOCE of BG (a), CBH (b), LAP (c), and AP (d) with soil microbial necromass AC and soil organic carbon (SOC). The curves in the figure are quadratic function curves. Treatment codes are shown in Figure 1.

The microbial necromass AC in the grassland, scrubland, and forestland was higher than that in the grazing land (Figure 1a). Compared with the grazing land, restoring the land to grassland and scrubland decreased the SOCE of BG by 34.2 and 29.7%, CBH by 27.1 and 37.9%, LAP by 41.4 and 25.2%, and AP by 42.7 and 25.5%, respectively, whereas restoring the land to forestland increased the SOCE of NAG by 61.1% (Figures 1b–f). The SOC content showed a quadratic relationship with the SOCE of BG, LAP, and AP, but a linear relationship with that of CBH. Moreover, no significant correlation was observed between microbial necromass AC and SOCE (Figures 2a–d).

### 3.2 Dynamics of MBCE

**Figure 3:**
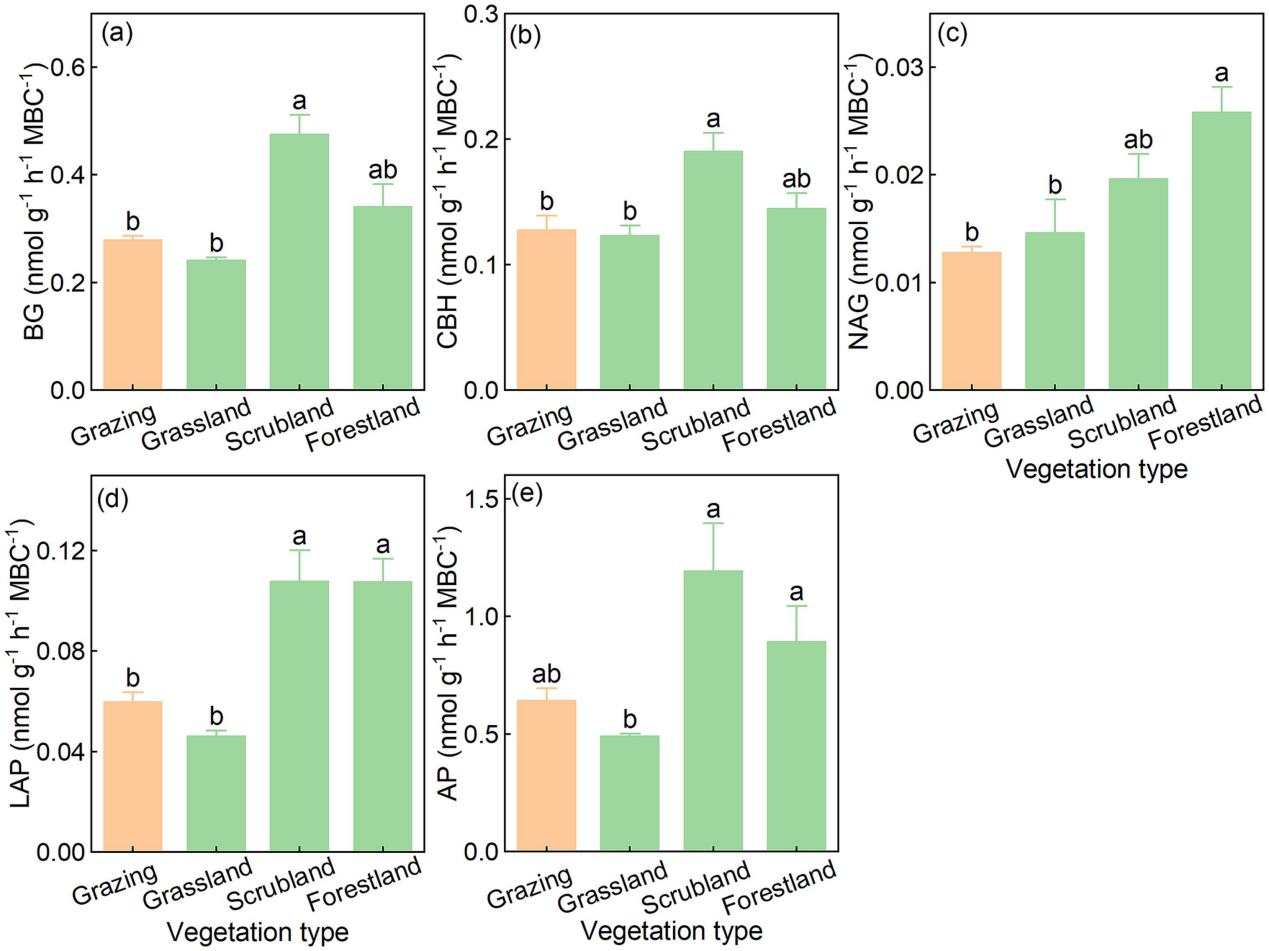
Effects of vegetation restoration on MBCE of BG (a), CBH (b), NAG (c), LAP (d), and AP (e). MBCE, soil-specific enzyme activity per unit of soil microbial biomass carbon. Treatment codes are shown in Figure 1.

**Figure 4:**
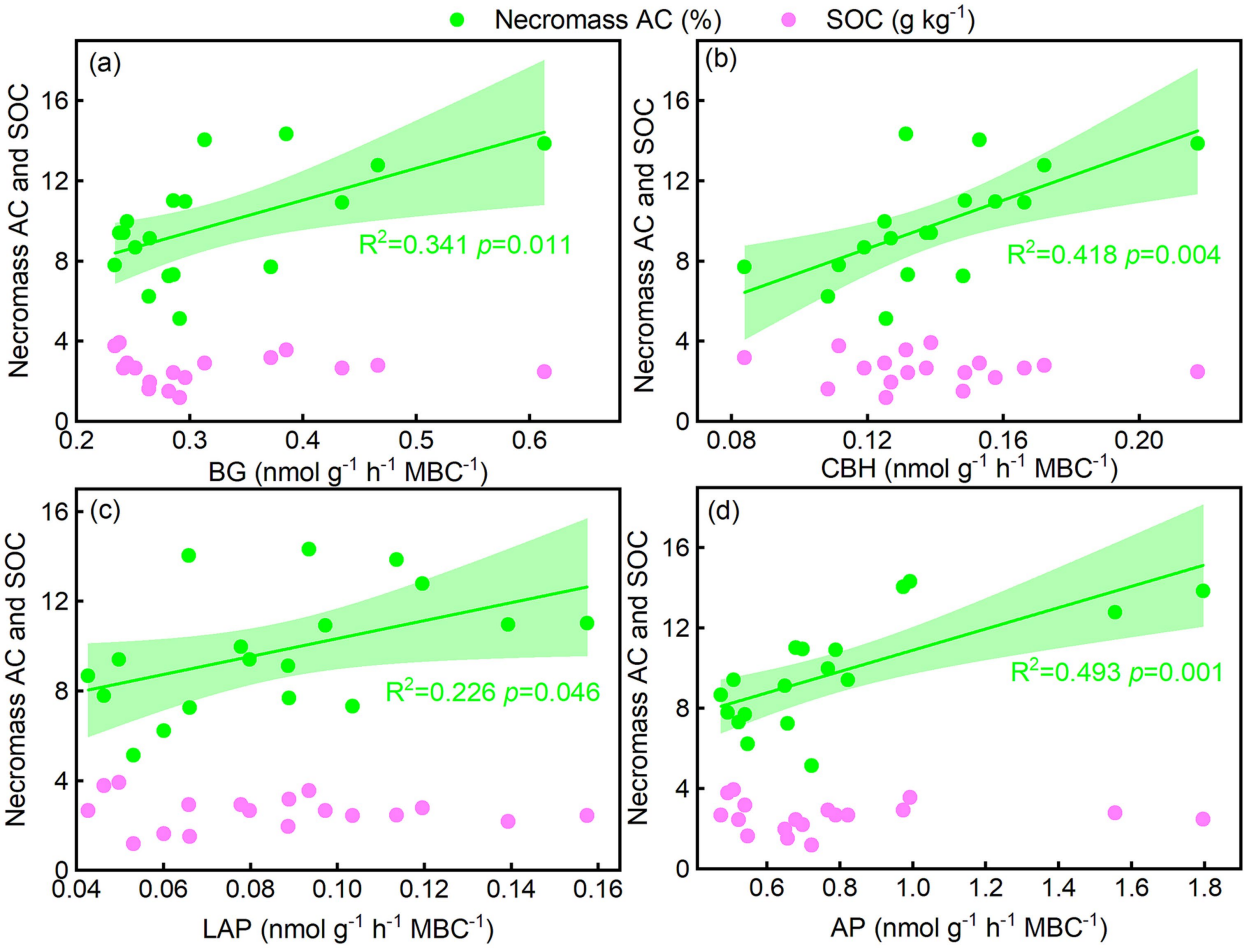
Regression relationships of MBCE of BG (a), CBH (b), LAP (c), and AP (d) with soil microbial necromass AC and soil organic carbon (SOC). Treatment codes are shown in Figures 1, 2.

Compared with allowing grazing, restoring the land to scrubland and forestland increased the MBCE of BG by 70.5 and 22.2%, CBH by 49.2 and 13.5%, NAG by 53.3 and 101.8%, LAP by 80.3 and 80.1%, and AP by 85.9 and 38.9%, respectively, whereas restoring the land to grassland decreased the MBCE of AP by 23.4% (Figures 3a–e). The necromass AC exhibited a significant positive correlation with MBCE of BG, CBH, LAP, and AP, whereas there was no significant correlation between SOC and MBCE (Figures 4a–d).

### 3.3 Dynamics of GMEA and soil enzyme activity coefficient

**Figure 5:**
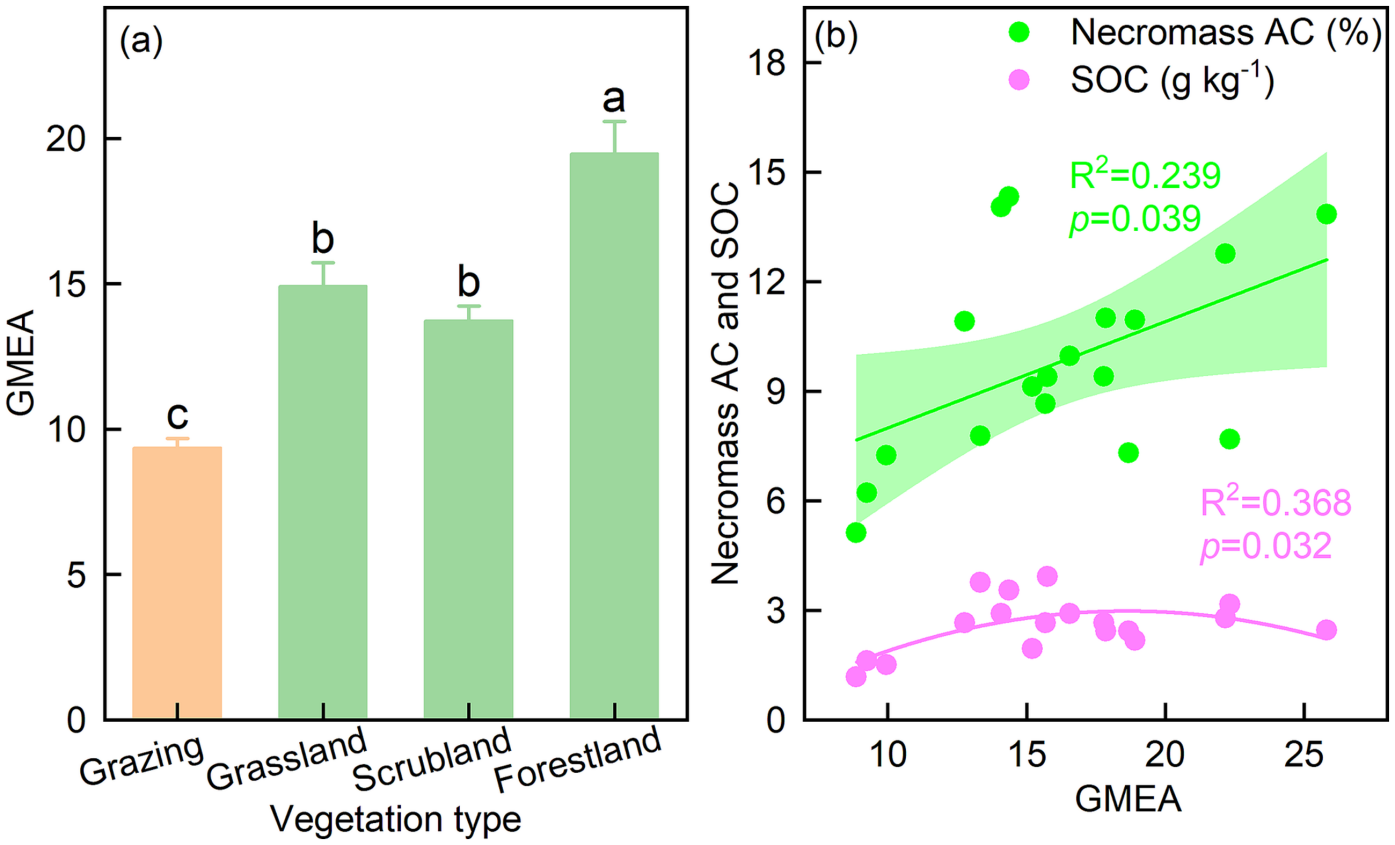
Effects of vegetation restoration on geometric mean of enzyme activity (GMEA) (a), and relationships of GMEA with soil microbial necromass AC and soil organic carbon (SOC) (b). Treatment codes are shown in Figure 1.

**Figure 6:**
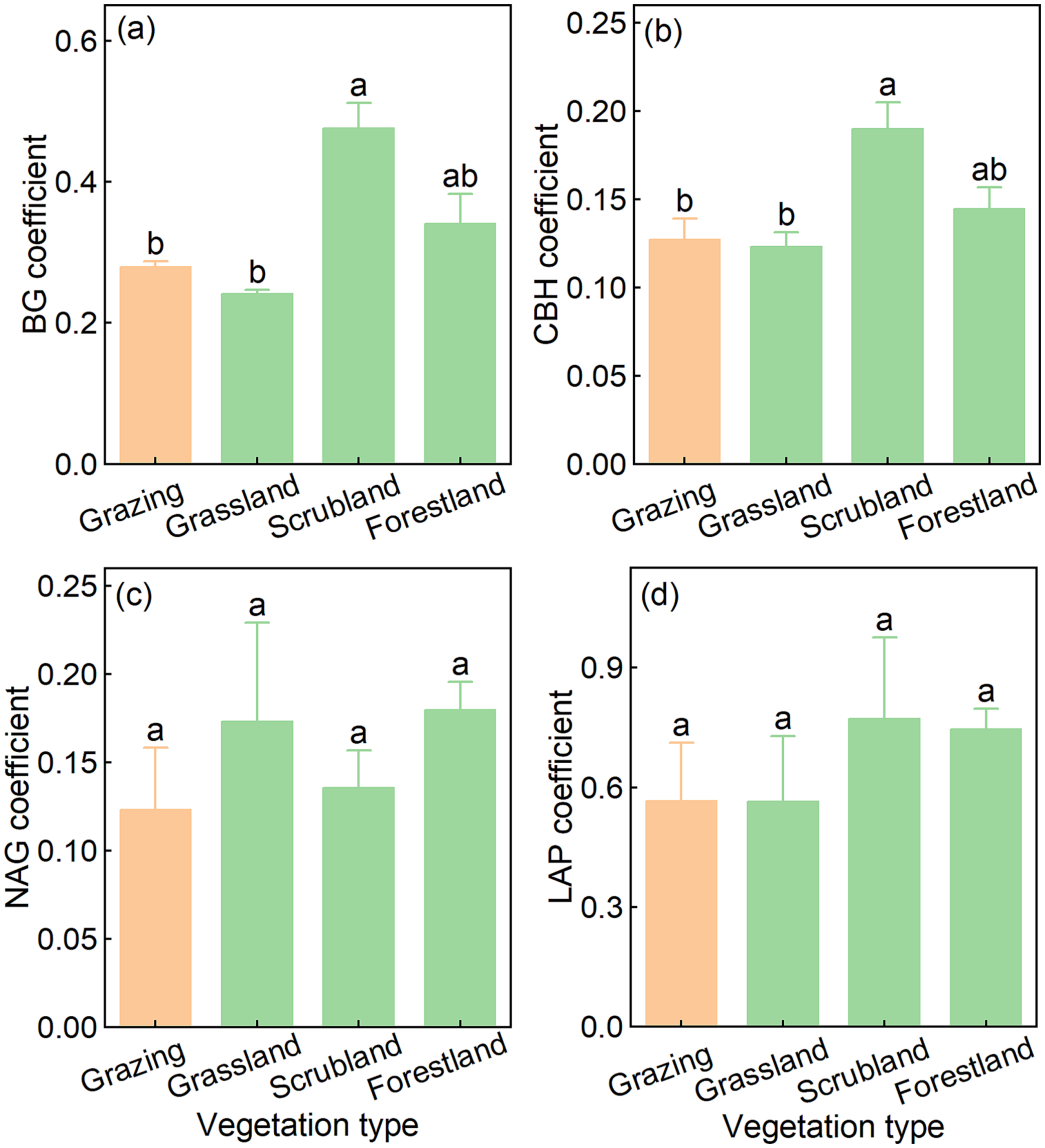
Effects of vegetation restoration on soil enzyme activity coefficients of BG (a), CBH (b), NAG (c), and LAP (d). Treatment codes are shown in Figure 1.

**Figure 7:**
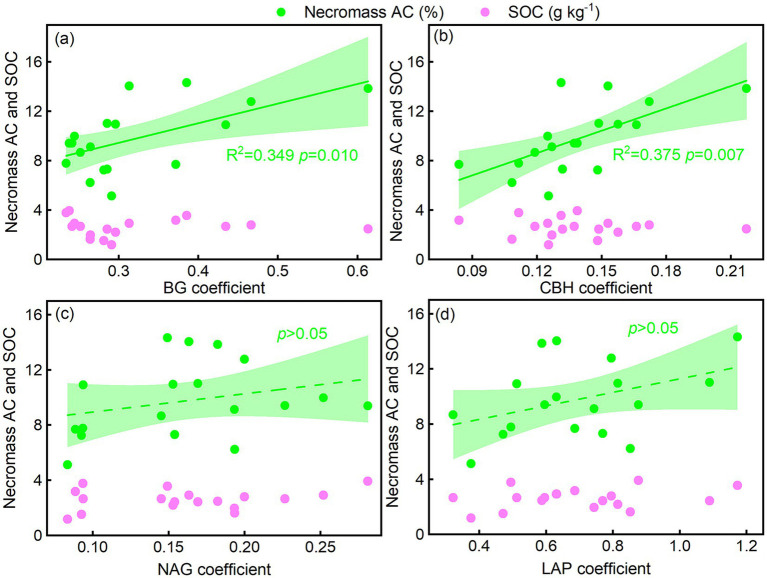
Regression relationships of soil enzyme activity coefficient of BG (a), CBH (b), NAG (c), and LAP (d) with soil microbial necromass AC and soil organic carbon (SOC). Treatment codes are shown in Figure 1.

Compared with allowing grazing, restoring the land to grassland, scrubland, and forestland increased the GMEA by 59.5, 46.8, and 108.2%, respectively (Figure 5a). The GMEA was linearly correlated with necromass AC but quadratic with SOC (Figure 5b). Compared with allowing grazing, restoring the land to scrubland and forestland increased the enzyme activity coefficients of BG and CBH, but no significant differences was observed in enzyme activity coefficients of NAG and LAP in grassland, scrubland, and forestland (Figures 6a–d). The enzyme activity coefficients of BG and CBH were significantly positively correlated with necromass AC, while NAG and LAP show no significant correlation with necromass AC (Figures 7a–d).

### 3.4 Factors affecting SOCE and MBCE

**Figure 8:**
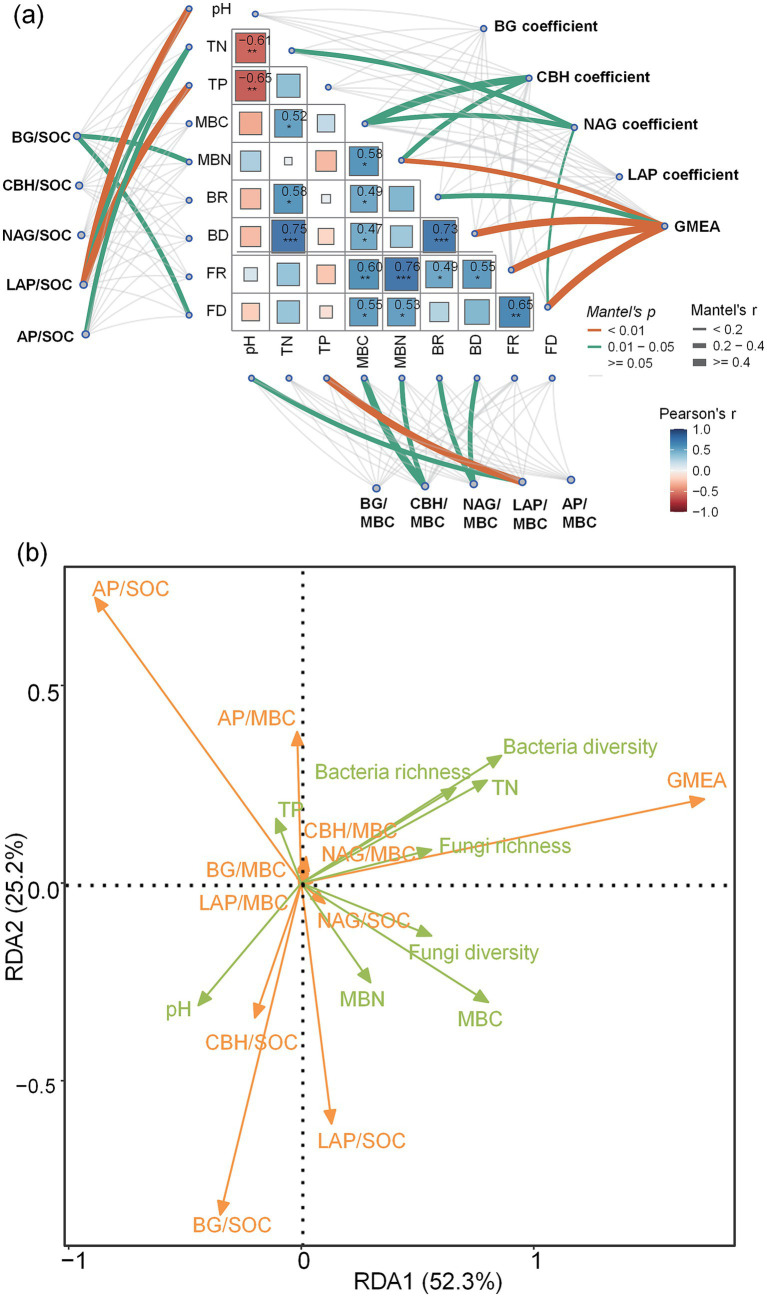
Relationships among soil physicochemical properties, bacteria diversity, fungi diversity, and soil-specific enzyme activity according to redundancy analysis (RDA) and Mantel tests. Treatment codes are shown in Figure 1. MBC, soil microbial biomass carbon; MBN, soil microbial biomass nitrogen; TN, soil total nitrogen; TP, soil total phosphorus; BD, bacteria diversity; BR, bacteria richness; FD, fungi diversity; FR, fungi richness.

Mantel test analysis revealed that the MBN and fungi diversity were the primary drivers influencing the SOCE of BG; the pH, TN, and TP were the key factors affecting the SOCE of LAP, while the TN was a key factor for the SOCE of AP. The MBC and MBN were major factors influencing the MBCE of CBH; bacterial community attributes (diversity and richness) were found to be the principal factors influencing the MBCE of NAG, while the pH was a key factor influencing the MBCE of LAP. The MBC and MBN were principal factors influencing the CBH coefficient; the TN, MBC, and fungi diversity were the main contributors to the NAG coefficient, while the MBN, diversity and richness of bacteria and fungi were key factors determining the GMEA (Figure 8a).

The RDA revealed a significant correlation between SSEA and soil properties, with the first two axes explaining 52.3 and 25.2% of the total variation, respectively. The SOCE of BG, CBH, NAG, and LAP were positively correlated with pH, MBC, MBN, and fungal diversity but negatively correlated with TN, TP, bacterial diversity, bacterial richness, and fungal richness. The MBCE of BG, CBH, NAG, and AP showed the opposite effects (Figure 8b).

## 4 Discussion

### 4.1 Effects of vegetation restoration on SOCE and its relationship with microbial necromass

Soil enzymes play an important role in promoting the transformation of soil matter and energy (Daunoras et al., 2024), and enzyme activity affects the availability of soil nutrients (Alkorta et al., 2003). Many studies have shown that vegetation restoration can provide soil microorganisms with available organic matter and abundant substrate (Graham and Knelman, 2023), thus promoting soil microorganism metabolism and reproduction and further improving the efficiency of microbial enzyme secretion (Sun et al., 2022; Araujo et al., 2024). Vegetation restoration can also regulate the soil physical structure and hydrothermal conditions (Lu et al., 2024). Soil temperature regulation and water retention ensure reasonable environmental conditions for soil enzymes during chemical reactions, thereby improving soil enzyme activity (Feng C. et al., 2019).

The GMEA can be used to characterize the microbial nutrient balance (Raiesi and Beheshti, 2014) and overall soil enzyme activity changes (Xu et al., 2020). As previously emphasized, ecological restoration increases soil enzyme activity (Feng Y. et al., 2019; Cui et al., 2020), and we also found that vegetation restoration increased the GMEA. However, restoring the land to grassland and scrubland decreased the SOCE of BG, CBH, LAP, and AP. Changes in the SOCE are mainly affected by SOC, enzyme activity, and microorganisms (Xu et al., 2020). Moreover, the RDA results showed that SOCE was positively correlated with MBC and MBN. Following ecological restoration, the improvement in soil structure and significant increase in soil microbial biomass and plant productivity (Dong et al., 2020; Liao et al., 2023) promoted SOC accumulation in the grassland and scrubland (Supplementary Table S1), but it had no significant effect on enzymes (Supplementary Figure S2), thereby reducing SOCE. In addition, SOCE was positively correlated with soil pH. Within a certain range, higher pH mitigates acid-induced limitations on microbial growth and activity, thereby facilitating microbial proliferation and metabolic activity (Hu et al., 2023). Our results further indicated that elevated soil pH is more conducive to microbial metabolic processes and enzyme synthesis. Notably, we found that SOCE was negatively correlated with TN, TP, bacterial diversity, bacterial richness, and fungal richness. This may be attributed to an increase in soil nutrients following vegetation restoration increases the soil microbial activity and diversity (Sha et al., 2023; Li Y. et al., 2024). However, an increase in the microbial biomass enhances their competition for soil resources (Llado et al., 2017), thus reducing their enzyme production efficiency (Wolejko et al., 2020).

Different vegetation types induce distinct alterations in soil physicochemical properties and nutrient availability (Lu et al., 2022). These shifts in the soil environment, in turn, drive soil microbial communities to secrete specific enzymes to meet their nutritional demands (Yin et al., 2014; Sha et al., 2023). In this study, the SOCE of NAG and LAP were higher in forestland than those in the grazing land. Soil enzymes primarily originate from microorganisms, plant and animal residues (Henry, 2012), and plant roots (Jin et al., 2024). Compared with grassland and scrubland, forestland has a higher litter amount (Wang X. et al., 2024), and with the decomposition of root secretions, nutrients are released and returned to the soil (Song et al., 2021), contributing to a higher microbial biomass in forestland (Supplementary Table S1). Simultaneously, the vegetation replacement rate was slow in the grazing plots (Sun et al., 2017), whereas forest restoration accelerated the growth of understory vegetation, thereby increasing soil microorganism activity and enzyme secretion. Our results indicated that with the restoration of understory vegetation, soil microbial biomass increased significantly, and more soil enzymes were generated to obtain organic matter to support intensive metabolic activities.

This study confirmed that vegetation restoration is an effective way to increase soil carbon accumulation (Supplementary Table S1). However, there were no significant correlations between the SOCE of BG, LAP, and AP and the necromass AC, possibly because microbial necromass carbon is mainly regulated by microbial community characteristics (Han et al., 2024). The relationships between GMEA and SOC, as well as SOCE and SOC, suggest that changes in GMEA and SOCE can be used as indicators of SOC accumulation. This is because rich soil nutrient conditions promote microbial growth, metabolic processes, and enzyme production (Zhan and Sun, 2014). High enzyme production requires high nutrient consumption (Wu et al., 2023) and enhanced microbial respiration (Gatica-Saavedra et al., 2023). Excessive SOCE may reduce SOC sequestration by increasing decomposition (Xu et al., 2020). However, when SOCE is high, the decomposition of litter and roots is substantially increased, thus improving microbial carbon utilization efficiency and promoting SOC accumulation (Li S. et al., 2024).

### 4.2 Effects of vegetation restoration on MBCE and its relationship with microbial necromass

We found that MBCE (BG, CBH, NAG, and AP) and GMEA had the same variation characteristics, both showing higher values in scrubland and forestland than in grazing land, confirming Hypothesis 1. The MBCE is often used to reflect the relationship between soil microorganisms and enzymes and the efficiency of soil microorganisms in producing enzymes (Xu et al., 2020). The results of our study indicate that soil microorganisms have a higher enzyme secretion ability and enzyme production efficiency when land is restored to scrubland and forestland. Changes in MBCE are regulated by soil microbial activity and nutrient contents (Raiesi and Beheshti, 2014). The RDA results showed that MBCE was positively correlated with TN, TP, bacterial diversity, bacterial richness, and fungal richness (Figure 8b). The increased availability of soil nutrients is conducive to the secretion of enzymes by soil microorganisms (Raiesi and Beheshti, 2015), which in turn increases MBCE. However, some studies have suggested that vegetation restoration reduces MBCE (Xu et al., 2020), which may be related to the differences in the sample sites selected in these studies. The sites selected in this study were arid areas with low rainfall, and the growth of plants and microorganisms was restricted by soil water content (Zhang et al., 2020). Water availability improves soil microbial community composition and promotes microbial growth and reproduction (Widanagamage et al., 2025). Therefore, microorganism activity and enzyme secretion are more susceptible to the effects of vegetation restoration. In addition, restoring the land to grassland had no significant effect on MBC and even reduced AP, indicating that although grassland had a higher MBC (Supplementary Table S1), it had a lower enzyme production efficiency. Therefore, from the perspective of microbial secretory enzymes, the vegetation restoration mode of grazing plots in this area should be preferentially selected as scrubland and forestland.

Microbial necromass accumulation is regulated by soil biotic and abiotic factors (Min et al., 2024). In this study, we found that, as biological factors of GMEA, the extracellular enzyme activity coefficient (BG and CBH) and MBCE were significantly positively correlated with necromass AC, indicating that a higher enzyme production efficiency of microorganisms was more conducive to the accumulation of microbial necromass. The study area is located on the Loess Plateau, which is an area with serious ecological degradation. Following grazing, the soil was compacted, plant growth was hindered, litter return was low (He et al., 2024; Wang K. et al., 2024), and soil microbial activity was low (Supplementary Table S1). In addition, the soil type in this region is eolian sandy soil with a shallow soil layer and a serious loss of soil water and nutrients (Zhang et al., 2020); thus, the soil nutrient content is low (Guo et al., 2023). In contrast, with the restoration of surface vegetation, plant diversity (Tian et al., 2023), soil microbial biomass (Liu et al., 2018), and bacterial and fungal diversity increased significantly (Huang and Shao, 2019), and soil nutrients were restored (Supplementary Table S1). Thus, soil enzyme activity increased, and the accumulation of microbial carbon was promoted. Simultaneously, following vegetation restoration, vegetation growth increases the quantity and quality of carbon input, and an increase in exogenous carbon improves the efficiency of the “microbial carbon pump” (Zhu et al., 2020). In addition, an increase in root carbon input leads to soil nutrients improvement, which provides more carbon substrates and energy for microorganisms, making the soil microbial biomass larger and more conducive to microbial growth and metabolism and the formation of microbial necromass (Hao et al., 2024). The results of this study emphasize that the GMEA, extracellular enzyme activity coefficient (BG and CBH), and MBCE can be used as important predictors of soil microbial necromass accumulation during ecological restoration.

## 5 Conclusion

SOCE and MBCE were sensitive to ecological restoration, but their responses differed markedly among vegetation restoration types. Restoring the land to grassland and scrubland decreased the SOCE, whereas restoring the land to scrubland and forestland increased the MBCE. Restoring to grassland, scrubland, and forestland increased the GMEA by 59.5, 46.8, and 108.2%, respectively. Meanwhile, the change in SOCE cannot be used to predict the change in microbial necromass carbon during vegetation restoration but can serve as an indicator of SOC, while MBCE is an important predictor of microbial necromass carbon. These results indicate that a higher enzyme production efficiency of microorganisms is more conducive to microbial necromass carbon. Moreover, from the perspective of MBCE, scrubland and forestland were preferred for vegetation restoration in sandy land areas. These results provide new insights into changes in soil enzyme activity during ecological restoration and a theoretical basis for vegetation construction, health management, and soil fertility enhancement in degraded ecosystems.

## Data Availability

The datasets supporting the conclusions of this article will be made available by the corresponding author, upon reasonable request.

## Footnotes

1
http://rhonin-bio.com/

2
http://www.baihuitech.cn/
