# Supplementary material for: Linkages between soil-specific enzyme activity and microbial necromass carbon: implications for ecological restoration
Source: Front Microbiol. 2026 Jun 12;17:1860192. doi: 10.3389/fmicb.2026.1860192 (PMC13303949; doi:10.3389/fmicb.2026.1860192)
Supplement: Supplementary file 2 [file Supplementary_File_1.DOCX]

Supplementary Material

# Supplementary Figures and Tables

## Supplementary Figures

**
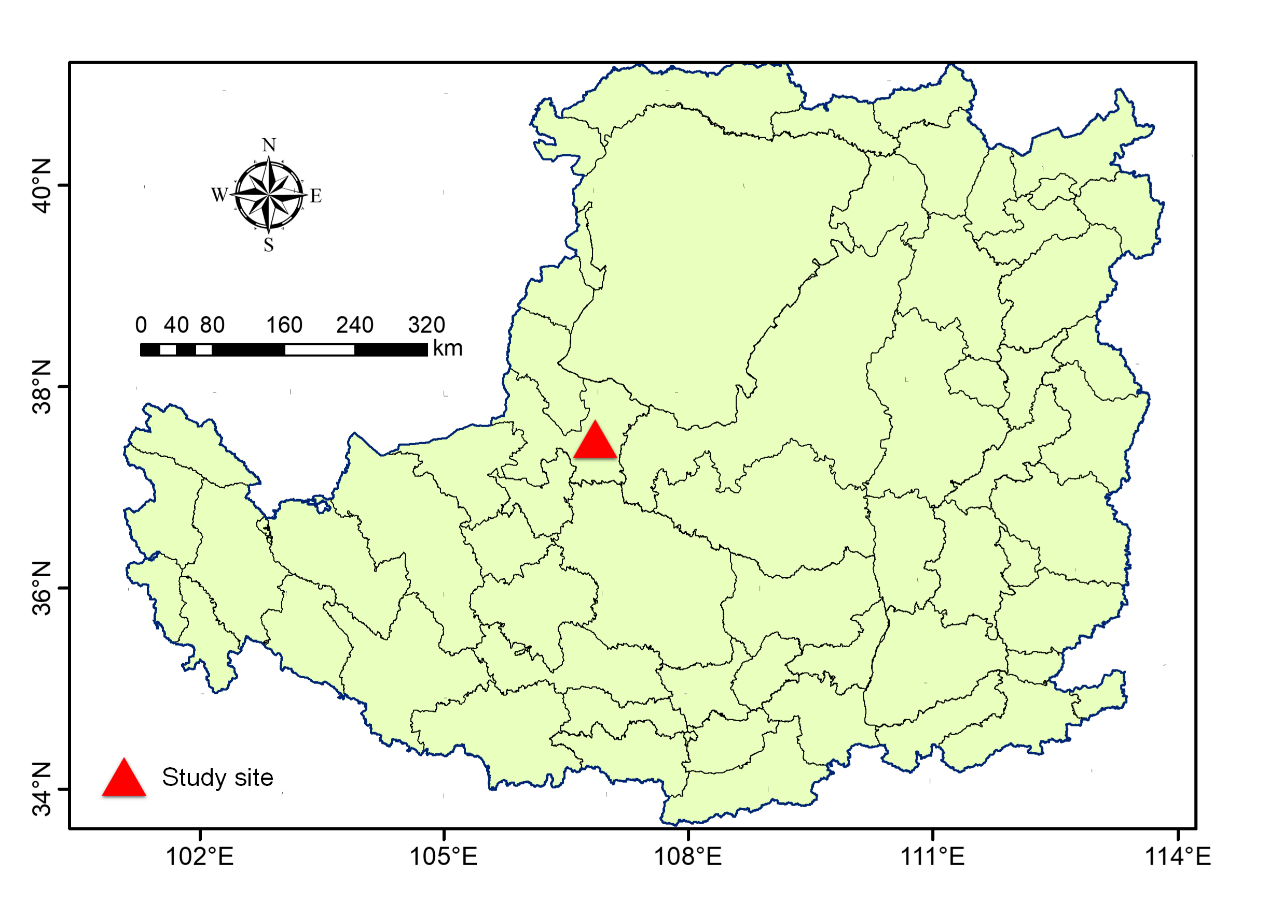
**

**Figure S1.** Geographical location of the study site.


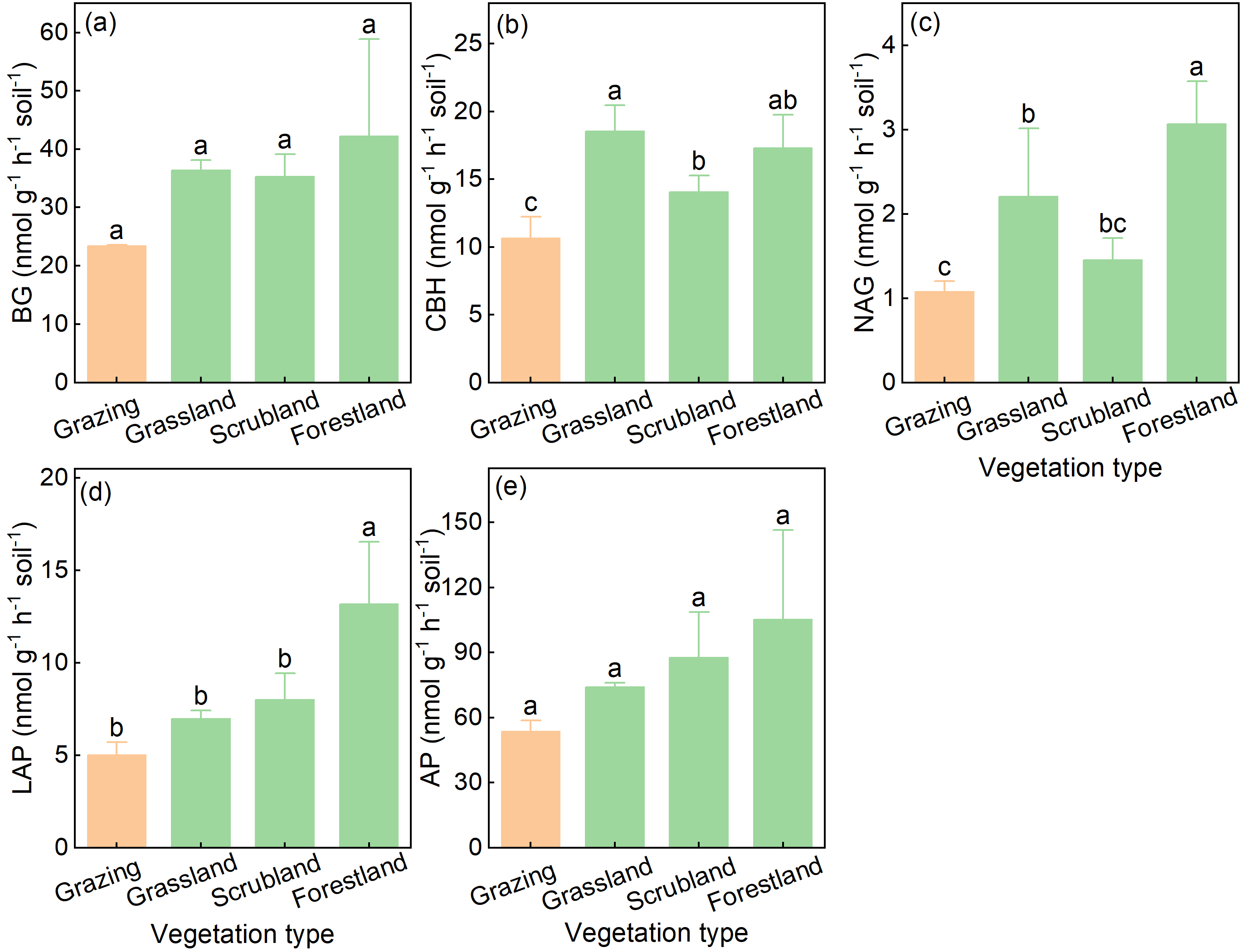


**Figure S2.** Soil absolute enzyme activity per unit of soil for (a) β-1, 4-glucosidase (BG), (b) β-D-cellobiosidase (CBH), (c) β-1, 4-N-acetylglucosaminidase (NAG), (d) L-leucine aminopeptidase (LAP), and (e) acid phosphatase (AP) in different vegetation types. Notes: Different letters indicate significant differences at *p* < 0.05.

- 1. **Supplementary Table**

**Table S1.** Soil physicochemical properties and soil microbial diversity of different vegetation types.

|  | pH | SOC (g kg^-1^) | TN (g kg^-1^) | MBC (mg kg^-1^) | MBN (mg kg^-1^) |
| --- | --- | --- | --- | --- | --- |
| Grazing | 8.81±0.03a | 1.43±0.13c | 0.10±0.00c | 83.99±2.24b | 9.76±1.78b |
| Grassland | 8.57±0.10b | 3.45±0.40a | 0.23±0.03a | 150.90±1.12a | 14.25±3.37ab |
| Scrubland | 8.68±0.04ab | 3.04±0.27ab | 0.16±0.01b | 74.54±3.11b | 11.32±2.12ab |
| Forestland | 8.79±0.03a | 2.55±0.12b | 0.15±0.01b | 124.96±10.09a | 17.80±1.27a |
|  | **Bacteria diversity** | **Bacteria richness** | **Fungi diversity** | **Fungi richness** |  |
| Grazing | 679.99±55.97c | 5.38±0.06b | 258.74±19.14c | 3.06±0.46a |  |
| Grassland | 1111.48±53.07a | 6.28±0.20a | 339.27±12.79ab | 3.85±039a |  |
| Scrubland | 791.33±28.29c | 6.02±0.08a | 301.21±19.77bc | 3.36±0.01a |  |
| Forestland | 965.49±32.31b | 6.16±0.04a | 373.01±16.60a | 3.92±0.15a |  |

Notes: Different letters indicate significant differences at *p* < 0.05.
